# The Effect of a ΔK280 Mutation on the Unfolded State of a Microtubule-Binding Repeat in Tau

**DOI:** 10.1371/journal.pcbi.1000155

**Published:** 2008-08-22

**Authors:** Austin Huang, Collin M. Stultz

**Affiliations:** Harvard–MIT Division of Health Sciences and Technology, Department of Electrical Engineering and Computer Science, Research Laboratory of Electronics, Massachusetts Institute of Technology, Cambridge, Massachusetts, United States of America; Indiana University-Purdue University, Indianapolis, Indiana, United States of America

## Abstract

Tau is a natively unfolded protein that forms intracellular aggregates in the brains of patients with Alzheimer's disease. To decipher the mechanism underlying the formation of tau aggregates, we developed a novel approach for constructing models of natively unfolded proteins. The method, energy-minima mapping and weighting (EMW), samples local energy minima of subsequences within a natively unfolded protein and then constructs ensembles from these energetically favorable conformations that are consistent with a given set of experimental data. A unique feature of the method is that it does not strive to generate a single ensemble that represents the unfolded state. Instead we construct a number of candidate ensembles, each of which agrees with a given set of experimental constraints, and focus our analysis on local structural features that are present in all of the independently generated ensembles. Using EMW we generated ensembles that are consistent with chemical shift measurements obtained on tau constructs. Thirty models were constructed for the second microtubule binding repeat (MTBR2) in wild-type (WT) tau and a ΔK280 mutant, which is found in some forms of frontotemporal dementia. By focusing on structural features that are preserved across all ensembles, we find that the aggregation-initiating sequence, PHF6*, prefers an extended conformation in both the WT and ΔK280 sequences. In addition, we find that residue K280 can adopt a loop/turn conformation in WT MTBR2 and that deletion of this residue, which can adopt nonextended states, leads to an increase in locally extended conformations near the C-terminus of PHF6*. As an increased preference for extended states near the C-terminus of PHF6* may facilitate the propagation of β-structure downstream from PHF6*, these results explain how a deletion at position 280 can promote the formation of tau aggregates.

## Author Summary

Alzheimer's disease pathology is characterized by two types of protein aggregates that are found in the brain—amyloid plaques and neurofibrillary tangles. Several studies suggest that these aggregates also play an active role in the disease process. Thus, an understanding of disease pathogenesis may be facilitated by a detailed characterization of the proteins that comprise these aggregates. Our study aims to model structural characteristics of tau protein, which is found in neurofibrillary tangles. Modeling of tau is particularly difficult because the protein is intrinsically disordered and therefore must be modeled as an ensemble of structurally dissimilar states. We developed a novel modeling approach that incorporates experimental measurements to generate ensembles of conformations that model the unfolded state of tau. By analyzing structural properties in these model ensembles for both normal and disease-associated forms of the protein, we identify structural features that may facilitate tau aggregation.

## Introduction

Alzheimer's disease (AD) pathology is characterized by extracellular aggregates of Aβ-amyloid (Aβ) and intraneuronal tau aggregates, known as senile plaques and neurofibrillary tangles (NFTs), respectively [1]. Despite much focus on Aβ amyloid in AD research, tau seems to play an important role as well. For example, the number of NFTs and not the number of senile plaques in the neocortex correlates with the severity of dementia in AD patients, and there are data that imply that abnormalities in tau alone may cause neurodegeneration [2]. In light of these observations, a detailed characterization of the structure of tau protein may provide insights into the pathogenesis of AD and other neurodegenerative disorders associated with tau pathology. However, probing the structure of tau is difficult because tau protein is natively unfolded (or intrinsically disordered) in solution. Several studies suggest that tau retains its function after heat or acid-induced denaturation and both CD and X-ray scattering experiments imply that tau does not adopt a well-defined folded structure in solution 3–5. Consequently, obtaining structural and hence functional information on tau is problematic because the direct observation of unfolded states is typically difficult to achieve experimentally.

Initially, unfolded proteins were described as random coils whose properties are derived from Flory's statistical description of chain molecules [6]. For such polymers, the radius of gyration, *R*_G_, follows the scaling law *R*_G_ = *R*_0_ *N^ν^*, where *R*_0_ is the radius of gyration of a monomeric subunit (a function of the persistence length), *N* is the number of subunits in the polymer, and *ν* is a scaling factor that depends on the solvent characteristics. The most common measure of whether a protein behaves as a random coil is to test whether its radius of gyration follows this scaling law. However, while a structurally disordered molecule can exhibit random coil statistics, the converse is not necessarily true; i.e., random coil statistics do not imply that the structure is completely disordered [7]. Slight structural preferences may exist for some natively unfolded proteins and small changes in the distribution of conformers within an unfolded ensemble may play a role in the normal and pathological functioning of intrinsically disordered systems. A recent study, for example, suggests that inducer-mediated tau polymerization involves an allosterically regulated conformational change [8]. This is consistent with the notion that the formation of tau fibrils is associated with a shift in the conformational distribution of tau such that the unfolded state has a preference for proaggregatory conformations in the presence of an inducer. In light of this, constructing detailed ensembles that model the unfolded ensemble of tau may facilitate the identification of structural properties that promote aggregation.

As full-length tau contains more than 400 amino acids (441 residues for the htau40 isoform [9]) constructing detailed ensembles that model the unfolded state of this protein is a daunting task. Fortunately, tau contains three or four imperfect microtubule-binding repeats (MTBRs) near the C-terminus of the protein, and almost all known mutations of tau that are associated with inherited forms of neurodegenerative diseases are located in MTBR domains or their nearby flanking regions [10]. As these data suggest that MTBRs play an important role in the progression of inherited tauopathies, we first focus on building ensembles that model the structure of individual MTBRs. It is important to note, however, that we do not strive to model the structure of a given MTBR fragment alone in solution. Rather, our goal is to generate ensembles that model the range of conformations that a MTBR can adopt when it is part of full length tau. In the present study we focus on building ensembles for the second MTBR, henceforth referred to as MTBR2. This repeat is of particular interest because it contains both a six amino-acid repeat, PHF6*, which is a minimum interaction motif that can initiate tau aggregation in vitro [11],[12], and the site of the proaggregatory mutation, ΔK280, which is associated with some forms of frontotemporal dementia [13]–[16].

We have developed a method, called energy-minima mapping and weighting (EMW), to construct ensembles that model the unfolded state of proteins. The underlying assumption that forms the basis of this approach is that the unfolded state can be modeled as a set of energetically favorable conformers, where each conformer corresponds to a local energy minimum. The method involves constructing a library of energetically favorable conformations and selecting conformations from this library to form ensembles that are consistent with a given set of experimental data. We use EMW to build ensembles for wild-type (WT) MTBR2 and the corresponding ΔK280 mutant. By comparing data from the two sets of ensembles, we deduce structural preferences in the ΔK280 ensemble that explain its increased propensity to form tau aggregates.

## Results

**Figure 1:**
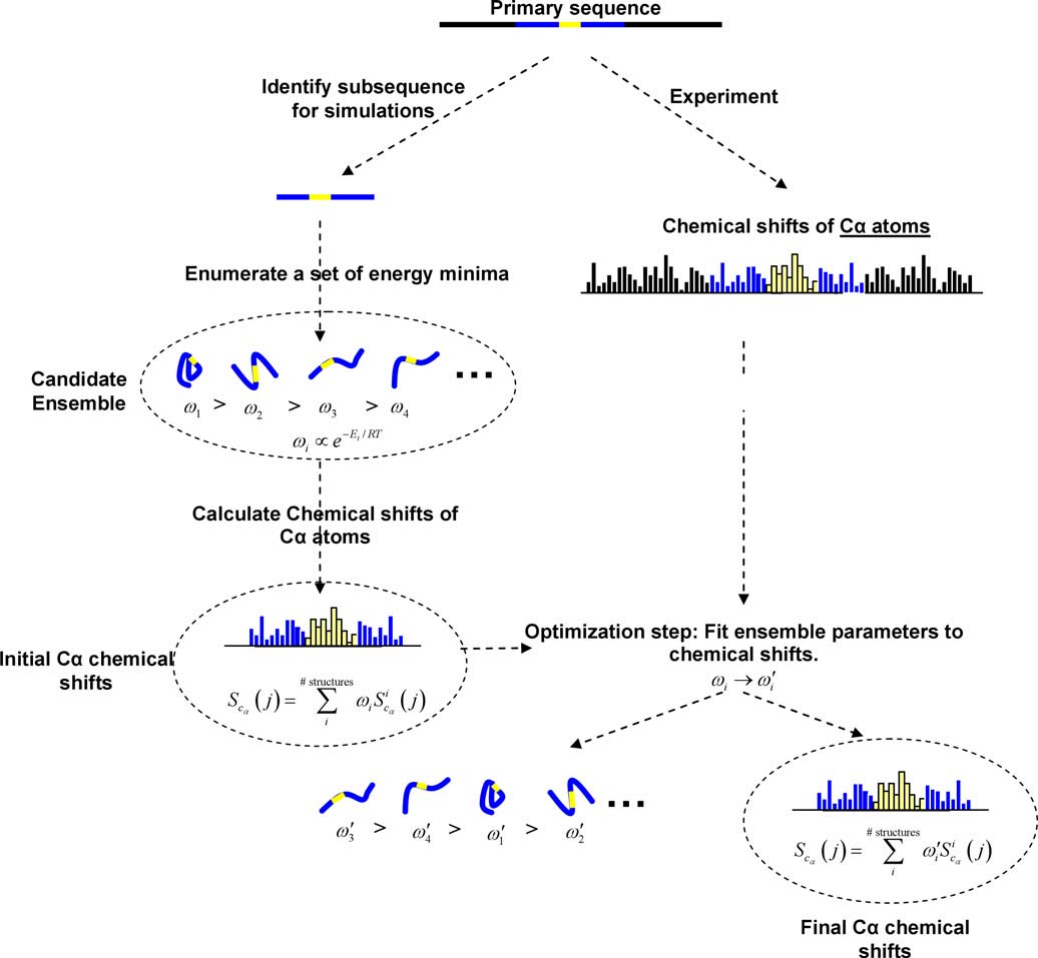
Outline of EMW method. The subsequence chosen for simulations is colored blue and contains an aggregation initiating sequence (colored yellow). A set of local energy minima can be enumerated using quenched molecular dynamics. Chemical shifts are calculated for the candidate ensemble and compared to chemical shifts obtained on the entire sequence. Weights of ensemble members are modified to improve agreement with experiment. 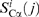 denotes the chemical shift of the Cα atom in the *j*th residue of the *i*th structure of the ensemble. 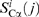 is computed from the *i*th structure using SHIFTX [20]. *S*_Cα_(*j*) is the statistical mechanical equivalent of the experimentally observed chemical shift of the Cα atom in the *j*th residue. We note that although the aggregation-initiating sequence is shown at the center of the chosen subsequence, this need not be the case. For MTBR2, the aggregation-initiating sequence is located at the N-terminus.

The EMW method begins by constructing sets of energetically favorable conformations for a sequence of amino-acids within a natively unfolded protein (Figure 1). In the case of tau we focus on MTBR2 since this region contains the aggregation-initiating sequence PHF6* as well as the site of a mutation that is associated with increased tau aggregation in vitro [17]. A set of local energy minima is then constructed for this subsequence, hence forming the candidate ensemble (Figure 1). Associated with each structure in this ensemble is a weight, *ω_i_*, which corresponds to the probability that the given subsequence adopts the *i*th conformation in the candidate ensemble. We say that an ensemble is fully specified when the local energy minima and weights are known.

Initial weights for structures in the candidate ensemble are calculated from the relative energies of each structure, as shown in Figure 1. However, as sampling is performed on a relatively small subsequence these weights may not reflect the relative probabilities of different conformations when the subsequence is part of the larger protein. For example, compact states may be preferred over extended states when the subsequence is in isolation but not when part of tau. Therefore, the composition of the ensemble is optimized and the members of the candidate ensemble are reweighted in light of experimental data that is obtained on a larger segment of tau protein. Sampling small subsequences increases the chance that we will observe a relatively large number of accessible states for this system. Using experimental data obtained on a larger region of tau (and not just the subsequence of interest) helps to ensure that the calculated ensemble represents the local structure of the sequence as it appears within full length tau.

A central component of EMW is that we do not strive to construct a single model for the unfolded state. We recognize that the construction of unfolded ensembles that agree with any given set of experimental data is largely an underdetermined problem; hence it is likely that there are a number of different ensembles that are consistent with a given set of experimental data. Consequently, we constructed several ensembles that are all consistent with the experimental measurements and focused our analysis on local structural motifs that are present in all ensembles. For this study, we focused on NMR data that are available for *both* WT MTBR2 and a ΔK280 mutant. These data were kindly provided by Marco Mukrasch, Daniela Fischer, and Markus Zweckstetter [17],[18].

Using the EMW method, 100 ensembles were constructed for both wild-type (WT) and ΔK280 sequences of MTBR2 (a total of 200 ensembles). Each ensemble was constructed to minimize the difference between calculated ^13^Cα chemical shifts and the corresponding experimentally determined ^13^Cα chemical shifts. The number of structures in each ensemble corresponds to the minimal number of structures needed to fit the available chemical shifts. Preliminary calculations found that 15 conformers were needed; i.e., fewer structures resulted in worse fits to the ^13^Cα chemical shifts and more structures did not significantly improve the quality of fits. We note that other models examining residual structure in the unfolded state have utilized a similar number of representative conformers [19].

**Figure 2:**
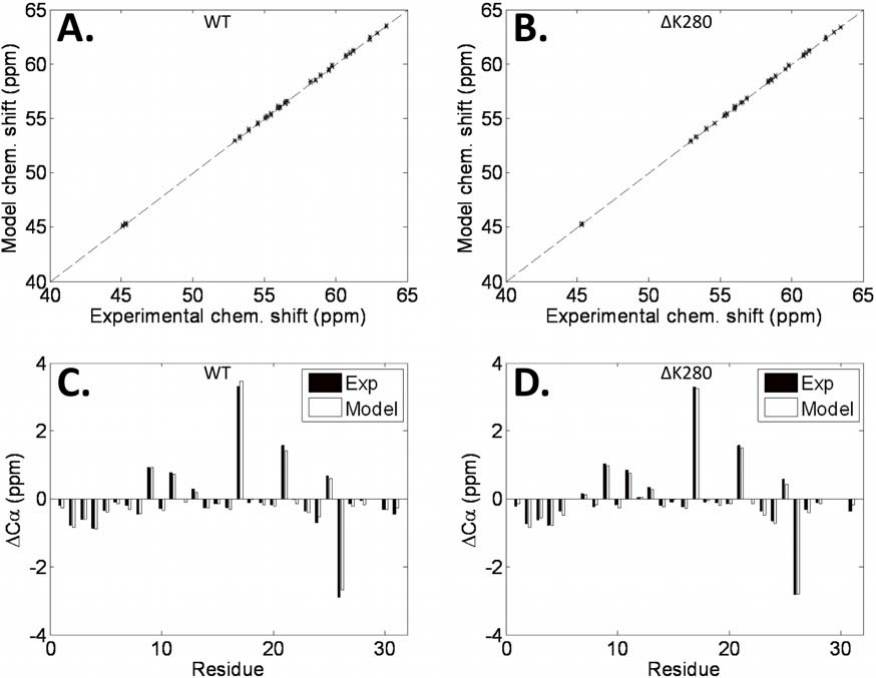
Model versus experimental absolute Cα chemical shifts and Cα secondary chemical shifts. Model versus experimental absolute Cα chemical shifts for (A) 100 WT ensembles and (B) 100 ΔK280 ensembles are shown. Cα secondary chemical shifts (ΔCα) are also shown for the (C) WT and (D) ΔK280 sequences using the ensemble that had the worst agreement with experiment. The worst model is defined as the ensemble that has the greatest RMSD between the calculated and experimentally determined values.

Application of EMW yielded ensembles that were in excellent agreement with experimentally determined absolute ^13^Cα chemical shifts (Figure 2A and 2B). The average RMS error between the calculated ^13^Cα chemical shifts and the corresponding experimental values was 0.1 ppm—well below the error associated with SHIFTX chemical shift predictions and similar to the error associated with experimental chemical shift measurements on K18 constructs [17],[20]. However, given that measured absolute chemical shifts for the 20 amino acids vary significantly according to the amino-acid type, reasonable correlations to absolute chemical shifts may be achieved by simply predicting amino-acid specific random coil values. Given this, we analyzed the relationship between the chemical shifts, after subtracting out residue-specific random coil chemical shift values; i.e., the secondary chemical shifts. Overall, there is excellent agreement between calculated secondary chemical shifts and the corresponding experimental values for each residue in the sequence (Figure 2C and 2D). These data demonstrate that the calculated models yield agreement with experiment on a per residue basis.

In the next step of our protocol, carbonyl carbon (^13^CO) chemical shifts were used to test whether the resulting ensembles can predict experimental observations that were not used to construct the model. This helps to ensure that our models are not “overly fit” to the ^13^Cα chemical shifts. In general, a model that is over-fit to a given set of experimental data can reproduce that data remarkably well but cannot reproduce data that was not used to generate the model. Therefore we consider an ensemble to be validated if new experimental results can be accurately predicted from the ensemble. For both the WT and ΔK280 sequences, each of the 100 ensembles was ranked based on its ability to predict ^13^CO chemical shifts. Based on these data the thirty best ensembles were chosen for further analysis. The RMS difference between the calculated ^13^CO chemical shifts and the corresponding experimental values are below 0.9 ppm; i.e., below the error associated with available chemical shift prediction algorithms (Table 1) [20]. To further demonstrate that these thirty ensembles can reproduce additional data not used in the model constructed, we computed the error between calculated amide hydrogen (^1^HN) chemical shifts and the corresponding experimental values. The resulting values agreed with the experimentally measured ones to within 0.3 ppm (Table 1).

**Table 1:** RMSD between calculated and experimental CO and H chemical shifts.

Ensemble	^13^CO		^1^HN	
	WT	ΔK280	WT	ΔK280
1	0.58	0.78	0.20	0.25
2	0.59	0.78	0.23	0.28
3	0.60	0.80	0.20	0.25
4	0.65	0.80	0.26	0.25
5	0.66	0.80	0.20	0.19
6	0.66	0.82	0.21	0.23
7	0.67	0.83	0.21	0.23
8	0.67	0.83	0.21	0.25
9	0.68	0.83	0.20	0.23
10	0.68	0.84	0.26	0.28
11	0.69	0.86	0.21	0.20
12	0.70	0.86	0.20	0.25
13	0.70	0.86	0.20	0.26
14	0.70	0.86	0.21	0.28
15	0.71	0.86	0.23	0.24
16	0.71	0.87	0.18	0.23
17	0.71	0.87	0.25	0.21
18	0.72	0.87	0.16	0.23
19	0.72	0.87	0.23	0.28
20	0.72	0.87	0.20	0.29
21	0.72	0.87	0.23	0.20
22	0.73	0.87	0.21	0.29
23	0.73	0.87	0.20	0.21
24	0.73	0.88	0.21	0.27
25	0.73	0.88	0.19	0.24
26	0.73	0.88	0.24	0.26
27	0.73	0.88	0.20	0.23
28	0.73	0.88	0.19	0.25
29	0.73	0.89	0.21	0.24
30	0.74	0.89	0.22	0.27

**Figure 3:**
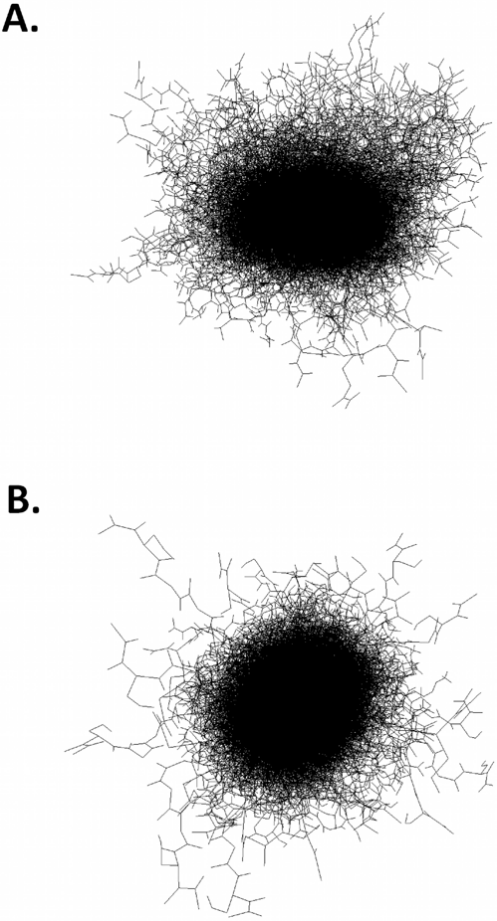
An alignment of structures from (A) all 30 WT ensembles and (B) all 30 ΔK280 ensembles.

As expected, structures that comprise the WT (Figure 3A) and ΔK280 (Figure 3B) ensembles are heterogeneous in that they sample a wide range of conformations. Since each of the 30 ensembles represents an independent representation of the unfolded state, we searched for local structural motifs that are found in all of the ensembles. More precisely, the existence of a local conformation that is consistently adopted by a given subsequence in MTBR2 suggests that this conformation is needed to reproduce the experimental results. We therefore consider conserved motifs to represent local conformational preferences.

**Figure 4:**
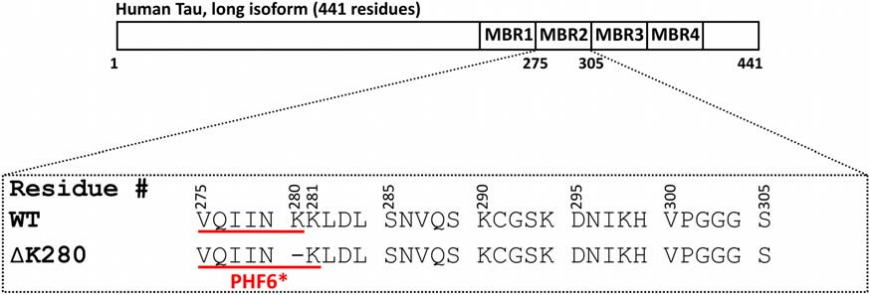
Aligned sequences of WT and ΔK280 tau. The PHF6* region is underlined in red.

**Figure 5:**
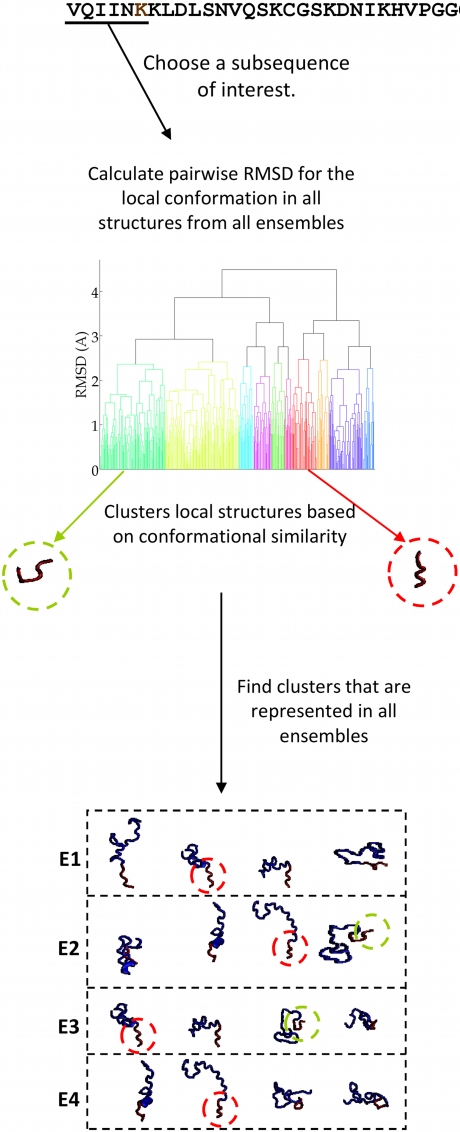
Outline of the method used for clustering local conformations. First, a six-residue local region is selected for analysis. Clusters of structures with similar conformations in the region of interest are formed based on pairwise RMSDs for backbone atoms in the local region. E1–E5 represent different ensembles. Clusters that are present in all model ensembles are circled in red, while clusters that are not preserved are circled in green.

We begin with an assessment of the local conformation of PHF6* in both the WT and ΔK280 ensembles. Since PHF6* in the WT sequence spans residues 275–280, the ΔK280 mutant sequence has a deletion in the six-residue stretch corresponding to PHF6*. However, since residue 281 is also a lysine, the ΔK280 mutant contains an equivalent PHF6* subsequence at its N-terminus (Figure 4). This allows us to directly compare the conformation of PHF6* in both sequences. To identify preserved conformations of PHF6*, we first determined the different types of structures that this subsequence can adopt by clustering structures using only the backbone atoms of PHF6* (Figure 5). The probability that a given cluster occurs in an ensemble is equal to the sum of the weights of structures in that ensemble that contains a motif in the cluster. Preserved structural motifs are defined as clusters that have a nonzero weight in every ensemble (Figure 5); i.e., a preserved motif is found in all ensembles. For comparison, we repeated this procedure for all contiguous six-residue subsequences within MTBR2, yielding a collection of approximately 300 clusters that represent all possible structural motifs in our ensembles that any six-residue sequence in MTBR2 can adopt. Using the criterion outlined above, roughly 5% of these clusters were preserved across all ensembles.

**Figure 6:**
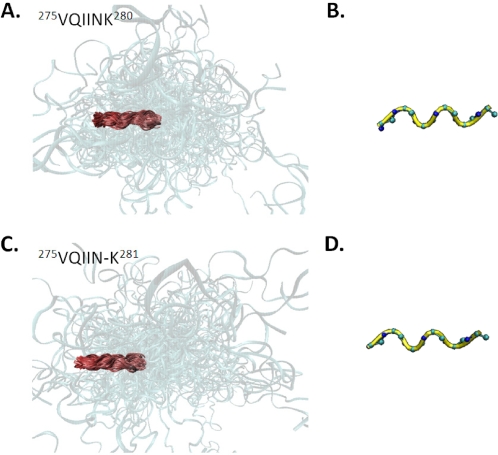
Structures of the cluster representing the local conformation of PHF6* that is preserved in all ensembles. (A) Aligned structures for WT tau and (B) average backbone conformation for this cluster; (C) aligned structures for the ΔK280 mutant and (D) corresponding average structure. The backbone of PHF6* is shown in yellow for the average structures.

In WT MTBR2, clustering based on the conformation of PHF6* yielded 12 distinct conformations. However, only one of these states was present in all 30 ensembles (Figure 6A and 6B). Similarly, while PHF6* clusters into 11 distinct conformations in the mutant ΔK280 ensembles, only one conformation was preserved (Figure 6C and 6D). In both cases, the preserved conformation of PHF6* is extended and has ϕ, ψ angles that fall within the broad region of the Ramachandran plot corresponding to β-structure. This observation is consistent with the notion that PHF6* a priori adopts extended conformations that can readily form cross β-structure with other tau monomers [21]. Since the formation of cross β-structure is believed to play an essential role in the formation of protein aggregates, these data are consistent with the notion that PHF6* promotes aggregation by forming β-structure between tau monomers [11],[12].

**Figure 7:**
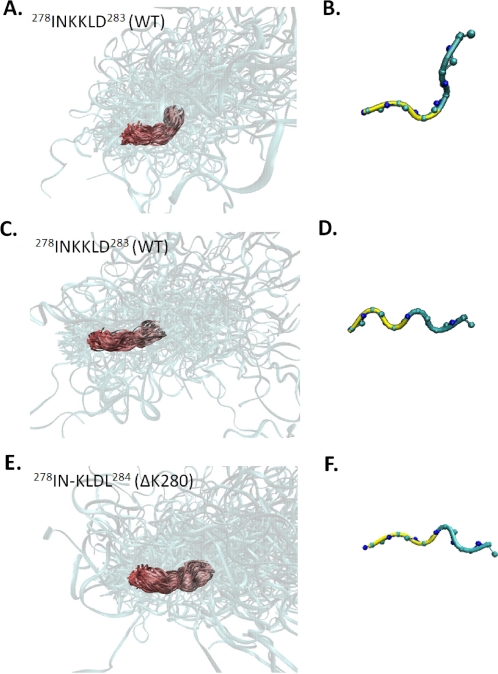
Preserved structures for the region corresponding to ^278^INKKLD^283^ in both the WT (A–D) and ^278^IN-KLDL^284^ ΔK280 ensembles (E,F). (A,C) Aligned structures corresponding to a preserved cluster in the WT ensembles aligned by backbone atoms of residues of ^278^INKKLD^283^ and (B,D) the corresponding average structures. (E) Aligned structures of the preserved cluster in ΔK280 ensembles, aligned by backbone atoms of residues ^278^IN-KLDL^284^. (F) The average conformation of the preserved cluster in ΔK280 ensembles. In the average structures, residues belonging to PHF6* are in yellow.

To explore the effect of the ΔK280 mutation on the local structure of MTBR2, we analyzed the structure of the subsequences ^278^INKKLD^283^ and ^278^IN-KLDL^284^ in the WT and ΔK280 sequences, respectively. For WT MTBR2, two conformations for ^278^INKKLD^283^ were found in all ensembles. The first is a loop/turn that is associated with a change in the direction of the mainchain (Figure 7A and 7B). In this structure residue K280 has ϕ, ψ angles of approximately −102° and −30°, respectively; i.e., mainchain dihedral angles consistent with an α-helical/turn conformation. The second conformation is more extended, having ϕ, ψ angles that place its residues within the broad region corresponding to extended β-structure (Figure 7C and 7D). In the mutant sequence, residue K280 is absent and the corresponding sequence, ^278^IN-KLDL^283^, has one preserved conformation. The deletion of residue 280, which can adopt an α-helical/turn conformation in the native sequence, leads to a relative increase in results in extended states in this region (Figure 7E and 7F). The deletion, however, also introduces a slight kink in the mainchain of the sequence (Figure 7F).

**Figure 8:**
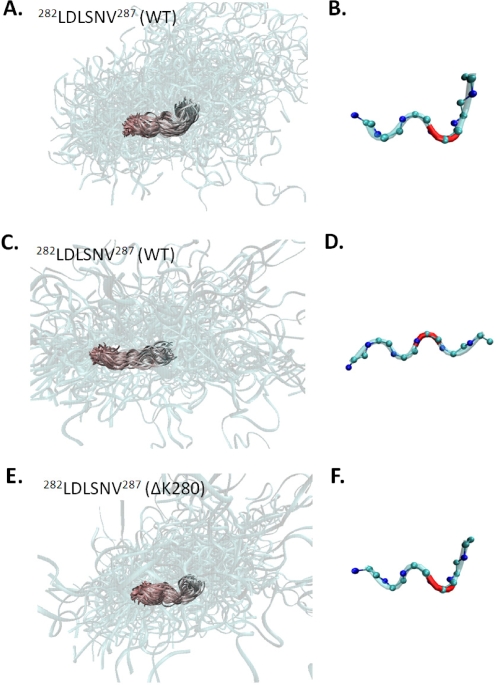
Preserved structures for the region corresponding to residues ^282^LDLSNV^287^ in both the WT (A–D) and ΔK280 ensembles (E,F). (A,C) Aligned structures corresponding to a preserved cluster in the WT ensembles aligned by backbone atoms of residues of ^282^LDLSNV^287^ and (B,D) the corresponding average structures. (E) Aligned structures of the preserved cluster in ΔK280 ensembles, aligned by backbone atoms of residues ^282^LDLSNV^287^. (F) The average conformation of the preserved cluster in ΔK280 ensembles. The location of S285 is shown in red in the average structures.

In a prior work, N–H residual dipolar coupling (RDC) values were measured for residues in the WT K18 construct in polyacrylamide gel [22]. While most residues in MTBR2 have relatively large negative RDC values, S285 has a large positive value [22]. This difference can be explained by either a change in the local alignment tensor at S285, or the presence of α-helical/turn structure at this site [23]–[26]. Accelerated molecular dynamics simulations of WT K18, however, confirm that the sequence ^283^DLSN^286^ samples turn conformations with relatively high frequency [22]. In light of these observations, we explored the structure of the six residue segment, ^282^LDLSNV^287^, which includes residue S285. This region adopts two conformations that are preserved across all WT ensembles. One of the conformations contains a loop/turn (Figure 8A and 8B) where residue S285 has ϕ, ψ angles of −63° and −39°, respectively; i.e., near the optimal α-helical values (Figure 8B). The alternate conformation is extended and does not result in a change in the direction of the mainchain (Figure 8C and 8D). However, in the ΔK280 mutant, ^282^LDLSNV^287^ has one structure that is preserved across all ensembles (Figure 8E and 8F). In this structure S285 again adopts ϕ, ψ angles (−95° and −63°, respectively) that are consistent with an α-helical/turn conformation (Figure 8F). These data agree with the RDC data mentioned above and suggest that this region in both the WT and mutant sequences is able to adopt turn-like conformations in solution as well as in a polyacrylamide gel.

## Discussion

Dynamical simulations provide a valuable tool for the analysis of unfolded proteins, providing insights that would be difficult to obtain from experiments alone [27]. A number of simulation methods have been developed to model the unfolded states of proteins and useful insights have been obtained with these techniques. Many of these approaches generate ensembles by directly incorporating experimental constraints into molecular dynamics simulations in order to facilitate conformational sampling. These methods bias molecular trajectories to sample conformers that are consistent with a given set of experimental data. One problematic issue with *biased sampling*, however, is that it can suffer from over-fitting—a process that may yield a distribution of conformers that does not accurately model the range of structures that comprise the unfolded state [27]. Given this concern, a number of *unbiased* methods have been developed to generate ensembles for unfolded proteins. These approaches utilize fast algorithms, which do not employ a physical potential energy function, to obtain representative structures of the unfolded state, and in some cases experimental data can then be used to improve the resulting ensembles [28]–[31]. The algorithm ENSEMBLE, for example, adjusts population weights for pregenerated conformers to improve agreement with experimental data in a manner similar to that described here [30].

A unique feature of the present method is that it does not strive to generate a single ensemble that represents the unfolded state. Given that accurate modeling of an unfolded protein is an undetermined problem, it is likely that there are a number of different ensembles that agree with any given set of experimental data. Moreover, given the immense number of potential conformations that an unfolded protein can adopt, this may be true even when a relatively large number of experimental constraints are used to construct the ensemble. Hence our goal was to construct several candidate ensembles, each of which agrees with a given set of experimental constraints, and focus our analysis on local structural features that are preserved across all ensembles. Local structural features that are found in all independent ensembles likely represent motifs that are required to reproduce the experimental data. In other words, given the underdetermined nature of the problem, it is not clear how to determine when one has the “correct” ensemble. However, local structural motifs that consistently appear in all independent ensembles are likely to also be present in the “correct” ensemble. Consequently, we consider locally preserved structural motifs to represent local conformational preferences.

An important consideration in our method is the choice of experimental data that is used to build and validate the constructed ensembles. In principle, EMW can use any set of experimental measurements to optimize and validate model ensembles. Indeed, as more structural information is made available, additional data can and should be used to further refine the set of model ensembles. In this regard, we note that although a number of NMR measurements have been made on native tau constructs, the data available for constructs containing a ΔK280 mutation is relatively limited. In a prior study, nuclear chemical shifts and HSQC spectra were measured for the K18ΔK280 construct, which contains all four MTBRs and the ΔK280 mutation [17]. Data were obtained for both free K18ΔK280 and for K18ΔK280 in the presence of the polyanion heparin and microtubules [17]. However, as we are interested in building structural models for MTBR2 in solutions free of compounds that promote tau self-association (e.g., heparin) and free of proteins known to bind tau, we focused on measurements obtained with the free K18ΔK280 construct. Additionally, as there are a number of existing methods that relate chemical shift measurements to three dimensional protein structures [20], [32]–[34] we considered ^13^Cα, ^13^CO, ^1^HN, and ^15^N chemical shift measurements; i.e., the only available chemical shifts for K18ΔK280 [17]. Furthermore, established methods for estimating NMR chemical shifts can predict carbon and amide proton chemical shifts with an error of approximately 1 ppm or less, while the error associated with predicting nitrogen chemical shifts is substantially larger (∼2–2.5 ppm) [20], [33]–[35]. Therefore we focused on the ^13^Cα, ^13^CO, and ^1^H chemical shifts for this study because these data represent measurements that can be calculated with the greatest accuracy and that are available for both native tau constructs and the ΔK280 mutant.

It has long been recognized that chemical shifts of a given residue are, in general, largely a function of the local environment of the residue in question [36],[37]. Since we generate ensembles that agree with chemical shifts, a limitation of the results reported here is that we do not explicitly include experimental data that more directly reveal information about non-local interactions. While long range contacts have been identified in some natively unfolded proteins (e.g., [19]), the dimensional scaling characteristics of intrinsically disordered proteins suggests that stable long-range contacts are sparse in these systems [38]. Nevertheless, we suggest that the combination of a physical potential energy function, which can in principle model long range interactions, and experimentally determined chemical shifts can provide insight into the structure of proteins in general. In this regard we note that data are emerging that suggest that backbone chemical shifts, when used in conjunction with a physical energy function, may be sufficient to adequately predict tertiary folds, and consequently stable non-local contacts, for some proteins [39],[40].

Although our work focuses on the structure of the MTBR2 without explicitly including other MTBRs, our findings may also have implications for full length tau. Once a representative set of conformers for MTBR2 is generated, we strive to ensure that the calculated chemical shifts agree with chemical shifts obtained using a construct that contains all MTBRs. This helps to guarantee that the ensemble models the structure of MTBR2 as it appears in full length tau. In short, we are not interested in the structure of MTR2 as it appears alone in solution; instead we hope to deduce structural features of MTBR2 as it appears in full length tau. In addition, as MTBR2 contains an aggregation-initiating sequence that is known promote tau aggregation in vitro as well as the site of a mutation that leads to in increased tau aggregation in vitro and in vivo, studies of both its WT and mutant forms may lead to insights into the mechanism of tau aggregation [12],[15],[41].

The ability to form intermolecular β-sheet conformations appears to be a relatively general property of polypeptide chains that are associated with disorders of protein misfolding and aggregation [42]–[45]. Therefore it is likely that an inherent propensity to form extended conformations, that are consistent with β-structure, will promote aggregation in natively unfolded systems. When EMW is applied to MTBR2, we find that the aggregation-initiating sequence, PHF6*, adopts an extended conformation in both the WT and ΔK280 ensembles, a finding consistent with the observation that these peptides can initiate tau aggregation [11],[12]. Interestingly, in a prior work we demonstrated that a related hexapeptide, PHF6, preferentially adopts an extended state that can facilitate the formation of cross-β-structure between tau monomers [21]. The present study suggests that this property is preserved when aggregation-initiating sequences are part of their corresponding MTBRs. That is, PHF6* a priori adopts extended conformations that can readily form hydrogen-bonded β-structure. Additionally, a recent survey of amyloidogenic proteins suggests that fibrillogenesis for natively unfolded proteins involve the formation of partially folded intermediates that can subsequently go on to form amyloid fibrils [45]. Our findings are consistent with these observations. That is, our results imply that formation of a locally stable, and extended, conformation plays a role in the formation of tau aggregates.

Recently, several studies have attempted to characterize residual structure of MTBRs in tau [17], [18], [22], [46]–[48]. These studies can be roughly divided into two categories: descriptions of ensemble average characteristics based on NMR measurements [17],[18],[22],[46], and NMR solution structures of local regions obtained by adding organic solvents to stabilize a unique fold [47],[48]. Since the presence of organic solvents leads to significant changes in the conformational distribution of states, as evidenced by dramatic changes in the CD spectra [5],[47],[48], the physiologic relevance of these latter results remains unclear. However, early characterizations of MTBRs in nonorganic solvents, found that the PHF6 region likely has a higher propensity for extended, β-strand-like conformations—a finding in accord with our data [18],[46].

Given that both WT and ΔK280 tau contain aggregation-initiating sequences (Figure 4), it is not clear how β-strand propensity in this region explains the difference in aggregation potential between the two sequences. Therefore to deduce structural features of the ΔK280 mutant that explain its proclivity to form aggregates, we analyzed the structure of MTBR2 in the vicinity of the mutation site. Unfolded ensembles of WT MTBR2 contain two conformations at the mutation site that were present in all ensembles—a loop/turn conformation and an extended state. In contrast to the WT MTBR2 ensembles, models of ΔK280 in the same region had one conformation that was present in all ensembles. This state is relatively extended and contains a kink at the site of the deletion. While the slight disruption in the extended state of the mutant may also influence the ability to form hydrogen-bonded cross-β-structure, a loop/turn at the C-terminus of PHF6* constitutes a much greater impediment to the formation of β-structure. Since residue K280 has a relative preference for nonextended states, deletion of this residue leads to increased sampling of extended states downstream from PHF6*. The relative preference for extended structures downstream from PHF6* in the ΔK280 mutant suggests that the ability to propagate β-structure distal to PHF6* can affect the aggregation potential of tau. These observations therefore explain how the deletion of a single residue can change the aggregation potential of tau.

We also find that in both WT and mutant ensembles residue S285 can adopt ϕ, ψ angles consistent with an α-helical/turn structure. Recent data on the WT sequence are also consistent with these observations as RDC values and molecular dynamics simulations suggest that S285 adopts an α-helical/turn structure. Since those experiments were performed in polyacrylamide gel, our data suggest that this structure also occurs with relatively high frequency in solution. It is also worthwhile to note that although we find that a six-residue region including K280 can adopt a similar loop/turn conformation, the associated RDCs for this region are not associated with a change in sign, like that observed at S285 [27]. Nonetheless, unlike RDC measurements for folded proteins, RDC values for unfolded proteins can be difficult to interpret [49]. This is due, in part, to the fact that prior to the measurement of RDC values, the protein of interest must first be embedded in an alignment medium [26]. This induced steric alignment of unfolded proteins may lead to results that do not fully capture the range of structures that an unfolded protein can adopt in solution. Hence the absence of particular RDC values in polyacrylamide gel (or any other alignment media) does not necessarily imply that a given conformation is not present in solutions containing the unfolded protein of interest.

The formation of tau aggregates is likely a complex process as a number of factors have been shown to influence the formation of tau aggregates in vitro [1]–[3]. Consequently, there may be additional factors that contribute to the increased ability of the ΔK280 mutant to form aggregates; e.g., a ΔK280 mutation leads to an overall decrease in the strength of the intermolecular charge-charge repulsion between tau monomers that self-associate [12]. Nonetheless, our data demonstrate that small changes in the sequence of tau can lead to localized structural changes in the unfolded ensemble that may affect tau's ability to form cross-β-structure. Overall, our data suggest that small sequence-specific changes can promote tau aggregation and that interventions that prevent the propagation of β-structure downstream from aggregation-initiating sequences, may form the basis for therapies that prevent tau aggregation.

## Methods

### Energy-Minima Mapping and Weighting

The EMW method constructs ensembles for unfolded proteins that are consistent with a given set of experimental data. Our model for an unfolded ensemble consists of structures corresponding to local energy minima and associated probabilities (weights) that are assigned to the different conformations. For this work, the experimental measurement used to optimize and validate the model ensembles are chemical shifts for the second tau microtubule binding repeat [17]. In principle, EMW can be used with any given set of experimental data. In this application we focus on chemical shifts that were available for both the K18 and K18ΔK280 constructs.

The EMW method can be decomposed into three steps (i) conformational sampling, (ii) model optimization, and (iii) ensemble validation. Conformational sampling uses high temperature molecular dynamics (MD) followed by minimization of the resulting structures (i.e., quenched dynamics) to create a library of widely varying conformations representing minima on the potential energy surface. Model optimization is performed to select a subset of these structures and optimize weights that represent the relative prevalence of each structure. Validation is performed by computing additional chemical shifts that not used to construct the ensemble and comparing these data to experimentally measured carbonyl carbon shifts. In what follows we outline each step of the EMW method.

#### Conformational sampling

We used quenched molecular dynamics (QMD) to sample different local energy minima of the R2 peptide. Conformational sampling was performed on a blocked peptide with the sequence corresponding to the second microtubule binding repeat. A polar-hydrogen model of the WT (VQIINKKLDLSNVQSKCGSKDNIKHVPGGGS) and ΔK280 (VQIINKLDLSNVQSKCGSKDNIKHVPGGGS) MTBR2 peptides were constructed using CHARMM [50]. The N and C-termini were blocked using ACE and CBX residues defined in the effective-energy function-1 (EEF1) model [51]. This sampling procedure consisted of high temperature molecular dynamics (used to randomize the initial conformation of the protein) followed by quenched dynamics. To ensure that a wide range of conformations was sampled, constraints were imposed on the peptide for the high temperature and quenching steps. Specifically, conformational sampling was performed in a series of molecular dynamics simulations. In each simulation the end-to-end distance of MTBR2 was restrained to a pre-defined value; i.e., 3, 4, 5, …, 70 Å, where the end-to-end distance was defined as the distance between the Cα carbons on residue VAL1 and SER31 of the peptide. End-to-end restraints were used to ensure that both compact and extended states were sampled during the high temperature simulations. For each end-to-end distance, 4 ns of high temperature MD at 1,000 K was performed with the EEF1 implicit model of solvent [51]. All simulations employed a Berendsen thermostat to maintain the system temperature at the desired value [52]. Hydrogen bond lengths were held near their equilibrium values using SHAKE [53] and a 2 fs timestep was used. Coordinates were saved every 10 ps, yielding a total of 400 structures per end-to-end distance. This procedure was applied to both WT and ΔK280 sequences, producing a total of 27,200 structures for each sequence.

Each structure was then used to initiate a new MD trajectory which cools the system to 298 K over 40 ps of simulation by coupling the sampled system (including atom coordinates and corresponding velocities) to a Berendsen heat bath at 298 K. At the end of this cooling simulation, structures were minimized for 10,000 steps using the adopted basis Newton–Rhapson algorithm [50]. Restraints were removed for the minimization step to ensure that minima on the unbiased energy surface are sampled. Searching for minima in the vicinity of the randomized conformation by cooling and equilibration followed by minimization rather than simply performing direct minimization allows the structures to escape shallow local energy minima and find more stable states.

As the conformation of PHF6* is of particular importance, additional simulations were performed to ensure that a large range of PHF6* conformations were represented in the ensembles. Each additional simulation constrained the PHF6* radius of gyration to adopt a predefined radius of gyration (4–5.9 Å) while the restricting the end-to-end distance of MTBR2 to be near 9 Å. This was done because our initial data suggested that compact conformations of MTBR2 were relatively undersampled after early QMD simulations. In total 31,200 local energy minima were generated for the native polypeptide and 31,200 structures were generated for the mutant structure. We refer to this set as our *structure library*.

We note that no single structure in our structure library had calculated backbone chemical shifts that agreed with the corresponding experimental values. For example, amongst the 31,200 structures, we found one conformer that had a ^13^Cα chemical shift error of approximately 1 ppm (compared to the ensemble shift errors of 0.1 ppm). In addition, this structure had a ^13^CO chemical shift error of 2.3 ppm (compared to the ensemble CO errors which were all below 0.9 ppm).

#### Ensemble optimization

The optimization procedure strives to obtain ensembles that have calculated chemical shifts that agree with experiment. The function to be minimized is:
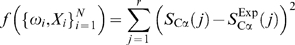
(1)where *N* is the number of structures in the ensemble, *X_i_* is the Cartesian coordinates of the *i*th structure, *ω_i_* is the weight of the *i*th structure, *r* is the number of residues in MTBR2, 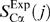 is the experimentally determined Cα chemical shift of residue *j*, and *S*_Cα_(*j*) is the calculated Cα chemical shift of residue *j*. Using the definition of *S*_Cα_(*j*) shown in Figure 1 we have:
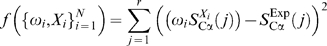
(2)where 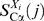 is the calculated chemical shift of residue *j* in structure *X_i_*. 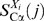 is computed using SHIFTX [20]. We note that reported errors for the experimentally determined chemical backbone shifts are all approximately 0.1 ppm [17]. Therefore, the experimental errors of individual shifts are not explicitly included in Equation 2. Lastly, errors reported in the text represent 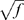 and are therefore in units of parts-per-million (ppm), i.e., the same units used for chemical shift data.

We used a simulated annealing algorithm to minimize *f* in Equation 2. To implement a simulated annealing protocol we first need an initial ensemble. The candidate ensemble was constructed by dividing the structure library into *n* different sets based on the radius of gyration of the different conformers (*n* was allowed to vary between 1 and >100, see below). One structure was randomly chosen from each set to form the initial ensemble. This ensures that our simulated annealing protocol begins with a set of structures that span many different radii of gyration for the molecule. The weights for structures in this ensemble were calculated from the relative energy of each conformation as follows:
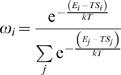
(3)where the energy associated with each conformation, *E_i_*, is the EEF1 potential energy, *S_i_* is the vibrational entropy, and *T* = 298 K [21]. This initial model (structures and weights) was the starting point of our simulated annealing protocol.

In our simulated annealing protocol, one performs a number of Monte Carlo steps at a given value of a control parameter (also referred to as the temperature). As the control parameter is gradually decreased, the system approaches its global minimum [54]. Central to any simulated annealing method is the protocol for decreasing the control parameter; i.e., the cooling schedule. We use a cooling scheduled based on the work of Nulton et al. and described in reference [55],[56].

Each Monte Carlo step consisted of several stages:

- Generating a new candidate ensemble:
  At each MC step, a structure from the current ensemble was replaced by a new structure from the library of minima (structure library) sampled by QMD to create a new candidate ensemble.
- Choosing weights for a given set of structures
  Given a new choice of *n* structures, weights were optimized using an minimization algorithm that employs an interior-reflective Newton method, to find a set of weights, *ω_i_*, which minimize [57],[58] Equation 2.
- Metropolis acceptance criteria
  The new ensemble (structures and weights) is accepted or rejected based on a Metropolis criterion.

The simulated annealing algorithm was implemented MATLAB (Mathworks). The number of Monte Carlo steps for a given value of the control parameter is as described in a previous work [55].

To determine the appropriate number of conformers in each ensemble, we performed the optimization procedure described above assuming that the ensemble had *n* structures, where n ranged from 1 to >100. These calculations found that a minimum of approximately 15 conformers were needed to fit the Cα chemical shifts to within 0.1 ppm, which is approximately equal to the experimental error associated with these chemical shift measurements [17] and well-below the error associated with SHIFTX chemical shift predictions [20]. Including additional structures did not significantly improve the error.

#### Ensemble validation

Validation consists of computing chemical shifts, using the final optimized model from, and comparing these data to experimentally measured values that were not used in step (ii). As described in the text, ^13^Cα-chemical shifts were used to construct the model and ^13^CO and ^1^HN shifts were used for validation purposes. The error between calculated and measured shifts is computed using Equation 2, with ^13^CO atoms substituted for ^13^Cα atoms. Models were ranked by their error and the 30 models with the best agreement with the ^13^CO shifts were selected for more detailed analysis as described in the text. To further test whether these models could be used to calculated quantities not used in model construction we computed ^1^HN chemical shifts from these thirty ensembles and compared these data to the corresponding experimental values.

### Identifying Locally Preserved Conformations

We searched for conformations of six-residue subsequences that are present in every ensemble. Six residues was a natural characteristic size for a local region of interest, as it is the length of PHF6*. To this end, all structures in each ensemble of either WT or ΔK280 MTBR2 were clustered using a matrix consisting of the pairwise RMSD backbone deviation of the each contiguous six-residue segment. Structures were clustered using MATLAB (Mathworks) such that the maximum RMSD between two structures in a cluster was 2.5 Å. A range of maximum RMSD values (1–6 Å) were examined empirically, and it was found that a cutoff of 2.5 Å was sufficient to prevent similar conformations from being divided into separate clusters, while also ensuring that clusters included a relatively homogeneous set of conformations. The probability that a given cluster occurs in an ensemble is equal to the sum of the weights of all structures that contain that motif. Preserved local structural motifs were found by identifying clusters where the total weight of its structures was non-zero across all ensembles.

Structures for each cluster were visualized in VMD. To facilitate visualization of the overall conformation associated with a cluster, an average structure for each cluster was generated after 5,000 steps of steepest descent minimization to remove bad contacts (only the 6 residues were minimized). Visual inspection verified that the energy minimized structures did not differ significantly from their un-minimized counterparts. All molecular structures were made with VMD [59].
